# Noninvasive assessment of asthma severity using pulse oximeter plethysmograph estimate of pulsus paradoxus physiology

**DOI:** 10.1186/1471-2466-10-17

**Published:** 2010-03-29

**Authors:** Donald H Arnold, Cathy A Jenkins, Tina V Hartert

**Affiliations:** 1 Departments of Pediatrics and Emergency Medicine, Vanderbilt University School of Medicine, Nashville, TN, USA; 2 Department of Biostatistics, Vanderbilt University School of Medicine, Nashville, TN, USA; 3 Department of Medicine, Division of Allergy, Pulmonary and Critical Care Medicine, Vanderbilt University School of Medicine, Nashville, TN, USA; 4 Center for Asthma Research, Vanderbilt University School of Medicine, Nashville, TN, USA

## Abstract

**Background:**

Pulsus paradoxus estimated by dynamic change in area under the oximeter plethysmograph waveform (PEP) might provide a measure of acute asthma severity. Our primary objective was to determine how well PEP correlates with forced expiratory volume in 1-second (%FEV_1_) (criterion validity) and change of %FEV_1 _(responsiveness) during treatment in pediatric patients with acute asthma exacerbations.

**Methods:**

We prospectively studied subjects 5 to 17 years of age with asthma exacerbations. PEP, %FEV_1_, airway resistance and accessory muscle use were recorded at baseline and at 2 and 4 hours after initiation of corticosteroid and bronchodilator treatments. Statistical associations were tested with Pearson or Spearman rank correlations, logistic regression using generalized estimating equations, or Wilcoxon rank sum tests.

**Results:**

We studied 219 subjects (median age 9 years; male 62%; African-American 56%). Correlation of PEP with %FEV_1 _demonstrated criterion validity (r = - 0.44, 95% confidence interval [CI], - 0.56 to - 0.30) and responsiveness at 2 hours (r = - 0.31, 95% CI, - 0.50 to - 0.09) and 4 hours (r = - 0.38, 95% CI, - 0.62 to - 0.07). PEP also correlated with airway resistance at baseline (r = 0.28 for ages 5 to 10; r = 0.45 for ages 10 to 17), but not with change over time. PEP was associated with accessory muscle use (OR 1.16, 95% CI, 1.11 to 1.21, P < 0.0001).

**Conclusions:**

PEP demonstrates criterion validity and responsiveness in correlations with %FEV_1_. PEP correlates with airway resistance at baseline and is associated with accessory muscle use at baseline and at 2 and 4 hours after initiation of treatment. Incorporation of this technology into contemporary pulse oximeters may provide clinicians improved parameters with which to make clinical assessments of asthma severity and response to treatment, particularly in patients who cannot perform spirometry because of young age or severity of illness. It might also allow for earlier recognition and improved management of other disorders leading to elevated pulsus paradoxus.

## Background

Clinicians have few objective measures to evaluate acute asthma severity and are likely to under-treat these episodes[1-6]. A severity measure should correlate with an accepted criterion standard (criterion validity) and quantify clinically important changes of this standard over time (responsiveness)[7].

Spirometry is the criterion standard for assessing the severity of airway obstruction (% predicted FEV_1_, %FEV_1_) but is effort dependent and not available in most acute care settings[8,9]. Airway resistance is another measure of lung function. Portable devices for measurement of airway resistance by the interrupter technique (Rint) are available and require only tidal breathing. Rint has been demonstrated to correlate with %FEV_1 _and specific airway resistance by body box plethysmography[10-13].

Accessory muscle use has been shown to be associated with a clinically meaningful decrease in %FEV_1_[14,15]. However, though accessory muscle use gives an indication of work of breathing, it does not provide a precise measure of airflow limitation.

Measurement of pulsus paradoxus (PP) during acute asthma exacerbations is currently recommended by national and international guidelines [16,17]. However, manual determination of PP is difficult, particularly in the tachypneic patient and in noisy clinical environments[18-21]. More than 98% of providers do not use this measurement at the bedside[22].

The pulse oximeter plethysmograph waveform closely mirrors radial artery Doppler waveforms, and previous investigations have provided evidence supporting the use of oximeter plethysmograph waveform data to estimate PP[23-28]. Developing methods to objectively quantify plethysmograph waveform data is clinically relevant as it might provide a non-invasive and continuous estimate of the severity of airway obstruction or other physiologic disturbances contributing to PP.

### Objective

Our primary objective was to determine how well a mathematic model for plethysmograph estimation of pulsus paradoxus physiology (PEP) correlates with %FEV_1 _(criterion validity) and change of this criterion standard (responsiveness) during treatment in pediatric patients with acute asthma exacerbations. Secondary objectives were to determine the correlation of PEP with airway resistance and accessory muscle use.

## Methods

### Study design and study population

We enrolled a prospective convenience sample of subjects ages 5 to 17 years with doctor-diagnosed asthma, signs or symptoms of an asthma exacerbation, (cough, dyspnea, shortness of breath, wheezing and/or chest pain),[29] and need for treatment with systemic corticosteroid (CCS) and inhaled albuterol as determined by the pediatric emergency medicine attending. The setting was an urban, academic, tertiary care children's hospital emergency department (PED). We excluded patients with pneumonia by clinical or radiographic criteria. Enrollment hours were 7 am to 10 pm weekdays and approximately every 3^rd ^weekend day. The Vanderbilt University Institutional Review Board approved the study protocol and a waiver of immediate informed consent such that baseline variables could be obtained prior to the informed consent process.

### Study protocol and measurements

All study data was collected by the principal investigator (DHA) or research assistant (DJR). The principal investigator trained the research assistant in the study protocol, and both individuals received training in portable spirometry performance by pediatric pulmonary function technicians. The investigators were not masked to study data during data acquisition.

At enrollment we recorded demographic information, medical history, family asthma history, asthma medication use, asthma symptom history, and Global Initiative for Asthma (GINA) chronic asthma control[16]. All other variables were obtained prior to administration of CCS (baseline) and bronchodilator treatments and again at 2 and 4 hours after CCS administration if the subject remained in the PED at that time.

**Figure 1:**
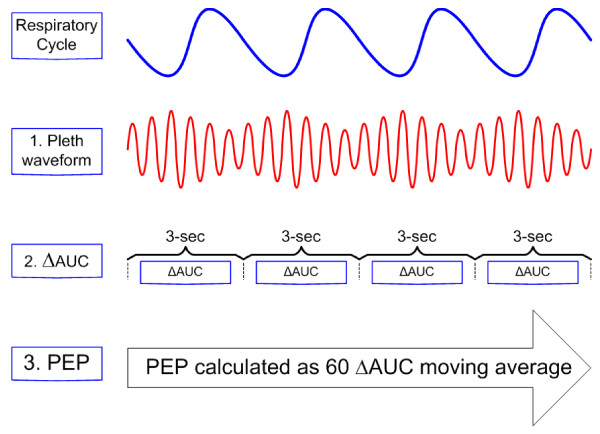
**Plethysmograph Estimate of Pulsus Paradoxus Calculation**. 1) AUC is calculated for each cardiac cycle from plethysmograph waveform. 2) ΔAUC is calculated for each successive 3-sec interval. 3) PEP is 180-sec moving average of ΔAUC.

PEP was determined at baseline, 2 and 4 hour time points as follows (figure 1)[30]. First, a Novametrix oxypleth pulse oximeter (Respironics Novametrix, Wallingford, CT) was configured to output the raw, high resolution waveform derived from the sensor's 940 nm infrared (IR) signal. We used this raw, unfiltered, unsmoothed signal in order to capture the full variability in waveform morphology during the respiratory cycle. This digitized IR signal was fed to a laptop computer via a serial cable and processed with a real-time, dedicated waveform analysis algorithm built for this purpose using graphical measurement and analysis software (LabVIEW 7.1, National Instruments, Austin, TX). Second, the plethysmograph waveform, constructed from the IR signal, was interrogated at 100 Hz. The software algorithm then calculated area under the curve (AUC) for each cardiac cycle as the area under the waveform bounded by the point of deflection from baseline to the point of return to baseline. During each successive 3-second time interval the smallest and largest AUC were identified and the percent AUC difference calculated (ΔAUC). Individual waveforms with AUC beyond 2 SD of a 180 second moving average were treated as artifact and excluded from further calculation. We chose the 3-second interrogation interval in an effort to include a full respiratory cycle because we anticipated that our subjects would have respiratory rates ≥ 20/min. Finally, the moving average of the 60 most recent ΔAUC (a 180-sec period) provided output of PEP.

We applied the oximeter sensor to a finger for PEP data acquisition. The subject was asked to keep the hand still and to not talk. This was necessary because the unprocessed IR signal is subject to movement artifact. We acquired PEP data over a minimum of 5 minutes to allow the graphical output of PEP to stabilize. The PEP value at the end of this period was recorded and is expressed as a percent value.

### Outcome measures

We used %FEV_1 _as the criterion standard to assess the diagnostic accuracy of PEP and used airway resistance and accessory muscle use as secondary severity measures. Accessory muscle use was defined as any visible use of the scalene, sternocleidomastoid, suprasternal, intercostal or subcostal muscles.

We measured airway resistance using a MicroDirect MRT6000 module (Micro Medical, Kent, England). This measurement was made prior to spirometry because the forced vital capacity maneuvers for spirometry can temporarily alter airway tone and airway resistance measures[31]. We applied a nose clip and instructed the subject to breathe comfortably while supporting the cheeks and submental tissue and extending the neck slightly[32]. Five measurements during exhalation were made and the median value recorded. The device calculates % predicted values for subjects ages 5 to 10 years (%Rint) using the McKenzie standards and outputs absolute values for subjects 11 years of age and above (aRint)[33].

We used a MicroDirect MicroLoop spirometer for %FEV_1 _determination. After applying a nose clip we instructed each subject to perform forced vital capacity maneuvers in accordance with American Thoracic Society (**ATS**) 1994 spirometry standards.^9 ^%FEV_1 _was calculated based on Knudson standards[34,35].

Some subjects could not perform 3 forced vital capacity maneuvers for each trial in accordance with ATS criteria because of the severity of acute asthma or young age. However, some of these trials included one or two maneuvers with acceptable flow-volume and volume-time curves.

A pulmonary function test oversight committee reviewed these non-ATS trials to determine if any of the data should be included in the analysis. This committee included a pulmonary physiologist and a pediatric pulmonary function lab technician (RRT). Each member recorded their determination whether a non-ATS trial should be retained for analysis based on the flow-volume and volume-time curves. Committee members were blinded to all other subject data and to the other member's determination. A non-ATS trial was retained for data analysis if both members independently determined that it should be retained.

### Sample size

We considered a correlation coefficient of 0.30 or greater to be clinically relevant based on the study of Wright and colleagues in which measurement of PP calculated from change in height of finger arterial pressure monitor waveforms was correlated with %PEF (r = - 0.31)[36]. A sample size of 82 subjects would enable us to detect a correlation coefficient of 0.35 or greater between PEP and %FEV_1 _values with 90% (β = .010) power and a two-sided significance level of 0.05 (α = 0.05). We set our sample size at a minimum of twice this calculated value in order to account for incomplete data. We anticipated that we would achieve the necessary sample size over a 12 month period and chose this enrollment period to minimize spectrum bias in seasonal asthma etiology and severity.

### Statistical analysis and data management

Descriptive statistics are presented as mean (SD) or median (IQR), as appropriate. We compared our study sample to the patient population ages 5 to 17 years seen in the PED during the study period with a final diagnosis code (ICD 493) of asthma exacerbation. This population data was extracted from a database designed primarily for billing purposes. While not directly comparable to our study sample, this data would provide a sense of how representative our sample was of the overall population meeting study inclusion criteria.

Differences, proportions, and correlations are reported as point estimates, bounded by 95% confidence intervals (CI). Analyses involving airway resistance are done separately for subjects ages 5-10 (%Rint) and ages 11 to 17 (aRint) years. The internal validity of PEP versus %FEV_1 _and PEP versus airway resistance at baseline was assessed using the Pearson product-moment correlation coefficient. The strength of the relationship between the proportionate change in PEP and the proportionate change in %FEV_1 _or the proportionate change in airway resistance was assessed using the Spearman's rank correlation coefficient. The relationship between PEP and accessory muscle use (present/not present) was examined in two ways. First, a logistic regression model using generalized estimating equations to account for repeated measures on a subject was used to assess the relationship between PEP and accessory muscle use, adjusting for time and any interaction it may have with changes in PEP. Second, Wilcoxon Rank Sum tests were used at each time point to determine whether the distribution of PEP values differ between those with accessory muscle use and those without.

**Figure 2:**
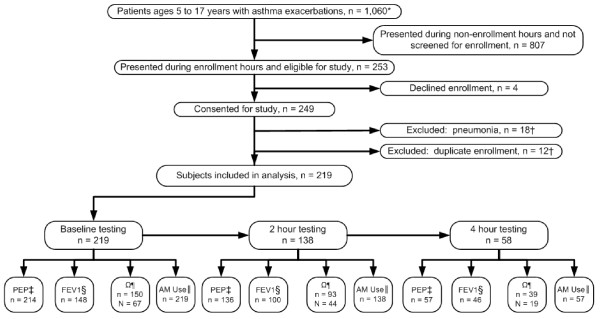
**Identification Study Subjects**.

All analyses were performed with R, version 2.8.1[37]. Study data were managed using REDCap (Research Electronic Data Capture) electronic database[38]. We have reported all 25 checklist items of the Standards for Reporting of Diagnostic Accuracy (STARD) initiative and have included a flow diagram (figure 2) to clarify subject recruitment and study implementation. Additionally, we included all data, including possible outliers, in the statistical analysis. We verified that the data met all assumptions for the statistical methods used.

## Results

### Descriptive statistics

During the enrollment period (June 8, 2008 to June 7, 2009), we recruited 249 subjects for this study, and 219 subjects are included for analysis (table 1 and figure 2). No subject experienced an adverse event as a result of study participation. Age, gender and race of subjects were similar to all patients ages 5 to 17 years of age who presented to our PED with an asthma exacerbation during the study period (table 1). Median respiratory rates (IQR) were 24/min (21-29) at baseline, 24/min (20-27) at 2 hours, and 24/min (21-26) at 4 hours. The numbers of subjects studied decreased at the 2 and 4 hour time points (table 2) as subjects were discharged to home or admitted to hospital. PEP improved between baseline and 2 hours (median 38% and 35%), in parallel with improvement in %FEV_1 _(median 61% and 69%), yet these improvements were not apparent in the group remaining at 4 hours (PEP median 38%; %FEV_1 _median 63%). Similar patterns are noted for airway resistance. These measures of lung function indicate that the subset of subjects remaining in the PED at later time points either improved more slowly or not at all.

**Table 1:** Subject and Study Population Demographic and Clinical Characteristics

Variable	PED Asthma Population* (n = 1,060)	Study Subjects (n = 219)
Age, median (IQR)	8.9 (6.9 - 11.7)	9.0 (6.9 - 11.9)
Male gender	625 (59)	135 (62)
Race†		
White	367 (34.6)	85 (39)
African-American	599 (56.5)	122 (56)
Hispanic	70 (6.6)	12 (5)
Other	24 (2.3)	0
Baseline PAS‡	NA§	5 (2 - 8)
Prior PICU admission	NA§	42 (19)
Prior endotracheal intubation for asthma	NA§	10 (5)
Disposition from pediatric ED	NA§	
Discharge to home		181 (83)
Admit to floor bed		25 (11)
Admit to PICU		13 (6)

Data are presented as the numbers (percentage) unless otherwise indicated.

*PED Asthma Population: all patients ages 5 to 17 years presenting to pediatric emergency department during study period with final primary diagnosis of asthma exacerbation by ICD code 493, including study sample.

†Race and Ethnicity for study subjects are categorized according to NIH notice NOT-OD-01-053. This does not include a category "Other."

‡Pediatric Asthma Score, Median (IQR)

§Not available in database used to identify PED asthma population

**Table 2:** Predictor and Outcome Variable Measurements

Variable	Baseline* (n = 219)	2 Hour* (n = 138)	4 Hour* (n = 58)
Predictor			
PEP†	38 (31 - 44, 214)	35 (29 - 40, 136)	38 (31 - 43, 57)
Outcomes			
%FEV_1_‡	61 (42 - 80, 148)	69 (52 - 82, 100)	63 (47 - 81, 46)
Airway resistance			
Age 5 to 10 yr‡	178 (142 - 241, 150)	143 (124 - 172, 93)	141 (124 - 161, 39)
Ages ≥ 11 yr§	0.71 (0.56 - 0.94, 67)	0.59 (0.44 - 0.75, 44)	0.68 (0.56 - 0.80, 19)
Access. M. Use¶	105 (48)	38 (28)	9 (16)

Data are presented as medians (IQR, number of non-missing values)

*Baseline value is before corticosteroid (CCS) administration. Two and 4 hour time points are times from CCS administration.

†PEP is Plethysmograph Estimate of Pulsus Paradoxus physiology. PEP values represent percent change in the plethysmograph waveform and have no unit values.

‡Percent predicted value

§ Absolute value (kPa/L/s)

¶Number of non-missing values (%) of subjects with any visible use of the scalene, sternocleidomastoid, suprasternal, intercostal or subcostal muscles

### Associations with outcome measures

**Figure 3:**
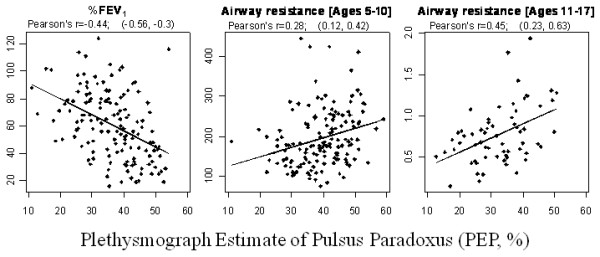
**Correlation of Plethysmograph Estimate of Pulsus Paradoxus with %FEV_1 _and Airway Resistance at baseline**. Horizontal axes in each panel are plethysmograph estimates of pulsus paradoxus (PEP) expressed as %. Vertical axes are percent predicted values for FEV_1 _and airway resistance (ages 5 to 10 years) and absolute value (kPa/L/s) for airway resistance (ages 11 to 17 years).

The correlations between PEP and %FEV_1 _and airway resistance are included in table 3 and are displayed graphically in figure 3. The confidence intervals for the correlations of PEP with %FEV_1 _at baseline and for proportionate changes over time do not span 0. These correlations indicate internal validity of PEP values with the primary criterion standard. Statistically significant correlations between PEP and airway resistance were noted at baseline but not for change of these variables over time.

**Table 3:** Correlations of Plethysmograph Estimate of Pulsus Paradoxus Physiology with %FEV_1 _and Airway Resistance

Outcome variable	Baseline* (r, 95% CI)	Baseline to 2 Hr change* (r, 95% CI)	Baseline to 4 Hr change* (r, 95% CI)
%FEV_1_†	-0.44 (-0.56, -0.30)‡	-0.31(-0.5, -0.09)	-0.38 (-0.62, -0.07)
Airway resistance			
Age 5 - 10 yr†	0.28 (0.12, 0.42)‡	0.06 (-0.16, 0.28) ¶	0.19 (-0.13, 0.51) ¶
Ages ≥ 11 yr§	0.45 (0.23, 0.63)‡	0.21 (-0.13, 0.52) ¶	0.25 (-0.24, 0.66) ¶

Values are Spearman rank correlation coefficient unless otherwise specified.

*Baseline value is before corticosteroid (CCS) administration. Two and 4 hour time points are times from CCS administration. Correlations at 2 and 4 Hr are for proportionate changes of PEP.

†Percent predicted baseline value and proportionate change of this value at 2 and 4 hr

‡ Pearson correlation coefficient (95% confidence intervals)

§Absolute baseline value (kPa/L/s) and proportionate change of this value at 2 and 4 hr

¶Numbers in parentheses are 95% confidence intervals derived using 1000 bootstrap replications.

A logistic regression model using generalized estimating equations was used to assess the relationship between accessory muscle use and PEP, adjusting for time. There was a statistically significant association of increased PEP and accessory muscle use (OR 1.16, 95% CI, 1.11 to 1.21, P < 0.0001). In addition, the model indicated that this association differed over time as seen by the significant interaction term (P < 0.0001). However, this result should be viewed cautiously given the small number of subjects who remained in the PED and had both 4 hour measurement of PEP and accessory muscle use at that time (n = 9). Wilcoxon Rank Sum tests were also used to assess the relationship of PEP with accessory muscle use at each time point. Test results for each time point across the groups were statistically significant at baseline (P < 0.0001) and 2 hours (P = 0.006), but not at 4 hours (P = 0.28). That this association was not statistically significant at 4 hours may be due to the small sample size at that time point (n = 57).

## Discussion

We found that the use of quantified pulse oximeter plethysmograph waveform data (PEP), a continuous, real-time and effort-independent measurement, correlates with %FEV_1 _(criterion validity, r = -0.44) as well as with proportionate changes in %FEV_1 _over the first 2 and 4 hours (responsiveness, r = - 0.31 and - 0.38) of acute asthma treatment in a PED. This compares favorably with the findings of Wright and colleagues who estimated PP based on change in waveform height and found correlation with %PEF (r = - 0.31)[36]. In our investigation we utilized %FEV_1_, the widely accepted criterion standard of acute asthma severity, in addition to secondary severity measures. We noted statistically significant correlations between PEP and airway resistance at baseline but not over time, and between PEP and accessory muscle use at baseline and over time.

There are limitations of this study. First, the three outcome measures may not fully reflect lung function, particularly in patients in significant respiratory distress. Spirometry is highly effort dependent. Although we ascertained the validity of each test by ATS criteria and review of flow-volume loops, some subjects may not have performed well due to respiratory distress or young age. Additionally, % predicted values of Rint for children ages 5 to 10 years of age are derived from a small sample (n = 236) of healthy children of four ethnicities and may not be a valid outcome measure[33]. Second, the raw, unfiltered, unsmoothed IR light signal must be used for waveform analysis in order to fully capture AUC variability that estimates PP. Movement artifact was possible, and signal stabilization methods might be employed to minimize this artifact. Third, outliers were noted (figure 3), possibly from movement artifact or individual differences in the physiologic events influencing PEP. The effect of these outliers was to decrease correlations of PEP with the outcome measures. Fourth, we programmed the software to calculate ΔAUC by identifying the smallest and largest AUC in successive 3-second time intervals. If this interval is shorter than the respiratory cycle, calculated PEP will underestimate PP; the inverse will apply if the interval is longer than the respiratory cycle. The former appears to apply overall because the median respiratory rates were 25/min at baseline and 24/min at 2 and 4 hours. We recognize the need to incorporate into this evolving technology a method for respiratory rate detection that will then allow gating the interrogation interval more precisely with the respiratory cycle. Lastly, there was very little change in PEP, %FEV_1 _and Rint from 0 to 2 to 4 hours. This may have been because those subjects remaining in the PED at 2 and 4 hours were minimally improved as the reason for not being discharged and, thus, had little change in PEP as a group.

## Conclusions

The results of this study indicate that PEP has criterion validity and responsiveness in pediatric patients with acute asthma exacerbations. Incorporation of this technology into contemporary pulse oximeters may provide clinicians improved parameters with which to make clinical assessments of asthma severity and response to treatment, particularly in patients who cannot perform spirometry because of young age or severity of illness. It might also allow for earlier recognition and improved management of other disorders leading to elevated pulsus paradoxus.

## Competing interests

DHA holds a patent related to the method of estimating pulsus paradoxus described in this study.

CAJ has no conflicts of interest to disclose. TVH has no conflicts of interest to disclose.

## Authors' contributions

DHA designed the study, enrolled subjects and is the primary author of the manuscript. CAJ conducted the statistical analysis and assisted in writing the *Statistical Analysis and Data Management*, Results and Discussion sections. TVH assisted with study design and assisted in writing the manuscript. All authors have read and approved the final manuscript.

## Authors' information

Dr. Arnold is Associate Professor of Pediatrics and Emergency Medicine, Monroe Carell Jr. Children's Hospital at Vanderbilt. Ms. Jenkins is Biostatistician III, Department of Biostatistics. Dr. Hartert is Associate Professor, Department of Medicine, Division of Allergy, Pulmonary and Critical Care Medicine. All authors are at the Vanderbilt University School of Medicine, Nashville, TN, USA.

## Pre-publication history

The pre-publication history for this paper can be accessed here:

http://www.biomedcentral.com/1471-2466/10/17/prepub
